# Experimental Bovine Spongiform Encephalopathy in Squirrel Monkeys: The Same Complex Proteinopathy Appearing after Very Different Incubation Times

**DOI:** 10.3390/pathogens11050597

**Published:** 2022-05-20

**Authors:** Pedro Piccardo, Juraj Cervenak, Wilfred Goldmann, Paula Stewart, Kitty L. Pomeroy, Luisa Gregori, Oksana Yakovleva, David M. Asher

**Affiliations:** 1 Laboratory of Bacterial and Transmissible Spongiform Encephalopathy Agents, Division of Emerging and Transfusion-Transmitted Diseases, Office of Blood Research and Review, Center for Biologics Evaluation and Research, United States Food and Drug Administration, Silver Spring, MD 20993, USA; piccardo43@gmail.com (P.P.); juraj.cervenak@fda.hhs.gov (J.C.); luisa.gregori@fda.hhs.gov (L.G.); oksana.yakovleva@fda.hhs.gov (O.Y.); 2 The Roslin Institute, University of Edinburgh, Midlothian EH25 9RG, UK; goldmannwg@blueyonder.co.uk (W.G.); pstewart18@sky.com (P.S.); 3 National Academy of Medicine of Uruguay, Montevideo CP 11200, Uruguay

**Keywords:** prion, TSE, bovine spongiform encephalopathy, squirrel monkey

## Abstract

Incubation periods in humans infected with transmissible spongiform encephalopathy (TSE) agents can exceed 50 years. In humans infected with bovine spongiform encephalopathy (BSE) agents, the effects of a “species barrier,” often observed when TSE infections are transmitted from one species to another, would be expected to increase incubation periods compared with transmissions of same infectious agents within the same species. As part of a long-term study investigating the susceptibility to BSE of cell cultures used to produce vaccines, we inoculated squirrel monkeys (*Saimiri* sp., here designated SQ) with serial dilutions of a bovine brain suspension containing the BSE agent and monitored them for as long as ten years. Previously, we showed that SQ infected with the original “classical” BSE agent (SQ-BSE) developed a neurological disease resembling that seen in humans with variant CJD (vCJD). Here, we report the final characterization of the SQ-BSE model. We observed an unexpectedly marked difference in incubation times between two animals inoculated with the same dilution and volume of the same C-BSE bovine brain extract on the same day. SQ-BSE developed, in addition to spongiform changes and astrogliosis typical of TSEs, a complex proteinopathy with severe accumulations of protease-resistant prion protein (PrP^TSE^), hyperphosphorylated tau (p-tau), ubiquitin, and α-synuclein, but without any amyloid plaques or β-amyloid protein (Aβ) typical of Alzheimer’s disease. These results suggest that PrP^TSE^ enhanced the accumulation of several key proteins characteristically seen in human neurodegenerative diseases. The marked variation in incubation periods in the same experimental TSE should be taken into account when modeling the epidemiology of human TSEs.

## 1. Introduction

Transmissible spongiform encephalopathies (TSEs or prion diseases) are a group of neurodegenerative diseases that affect humans and animals [1]. The most common TSE in humans is Creutzfeldt-Jakob disease (CJD) [1], which can be sporadic (sCJD), iatrogenic (iCJD), familial (fCJD, also termed “genetic” (gCJD)) or variant (vCJD) [2,3]. Bovine spongiform encephalopathy (BSE), recognized initially in the UK and later in more than 25 other countries, is the first TSE confirmed to be zoonotic—an infection of animals transmitted to humans exposed to the BSE agent through consumption of contaminated meat or meat products or, very rarely, to recipients of blood transfused from donors incubating the vCJD [2,3]. All TSEs are characterized by accumulations of a misfolded isoform (PrP^TSE^) derived from normal “cellular” prion protein (PrP^C^) in brain and sometimes in other tissues. PrP^C^ is a highly conserved protein of unknown function present in many organs of normal humans and animals. PrP-knockout animals engineered to lack PrP^C^ are not susceptible to TSEs or infection with TSE agents [4]. Initial results of several studies suggested that TSE agents infect mainly cells of the nervous system and lymphoid lineages [5,6]. Susceptibility of cells to infection with TSE agents cannot be reliably predicted from the tissue of origin or the expression levels of PrP^C^ [6]. Studies in vitro showed that, independent of cell type and TSE agent strain, PrP^TSE^ formed rapidly after infection of both neuronal and non-neuronal cells in culture, but that did not always lead to persistent infection of those cells [5,6]. Some unknown cellular or TSE-agent-specific factors are probably required to generate persistent TSE infections. Recent studies amplified protease-resistant PrP or fibrillary PrP-related peptides in vitro, allowing detection of very small amounts of PrP^TSE^ in tissues and some fluids of humans and animals with TSEs; those techniques could be used to study TSE pathogenesis and transmission, but are currently employed mainly as research tests to help diagnose human TSEs antemortem [7]. PrP^TSE^ amplification techniques yielded false positive results in rare instances [8] so that histopathological analyses remain necessary to confirm TSEs.

When agents derived from cases of vCJD originating in five different countries (Canada, Italy, The Netherlands, UK, USA) were inoculated into a panel of selected well-characterized wild-type mice, the animals developed TSEs with similar histopathological lesion profiles, immunohistochemical changes, and PrP^TSE^ banding patterns by Western blot (PrP “glycoforms”), supporting the hypothesis that a single strain of infectious agent (“classic” or C-BSE) caused all vCJD cases in North America and Europe and further suggesting that current clinical criteria for diagnosing vCJD are reliable [9].

The epidemic of BSE in UK cattle in the 1980s and 90s was followed by a relatively modest number of diagnosed cases of vCJD (233 total cases worldwide—all but 55 in the UK—as of May 2022, reported by the UK CJD Research and Surveillance Unit, Edinburgh [www.cjd.ed/uk], accessed on 12 May 2022), among tens of millions of people probably exposed to the agent. That observation raised questions about the overall transmissibility of the agent to humans and the possible existence of some unknown number of latent or asymptomatic cases of vCJD [2]. Experiments in monkeys—large long-lived animals genetically more closely related to humans than are rodents and ruminants—might help to address some of those issues. Studies in monkeys might also help to elucidate the possible role that aggregates of brain proteins other than PrP play in pathology and pathogenesis of various human TSEs [10]. As part of a long-term study investigating susceptibility of several widely used cell cultures to BSE infection, we inoculated squirrel monkeys (SQ) with serial dilutions of a bovine brain suspension containing the C-BSE agent and monitored them for ten years. In a preliminary study, we showed that SQ infected with C-BSE (SQ-BSE) developed a spongiform encephalopathy resembling that seen in humans with vCJD [10,11], albeit without PrP plaques. Here, we report the final characterization of this experimental model after observation over ten years. We observed an unexpected extreme difference in incubation times between two animals inoculated with the same dilution and volume of a C-BSE brain suspension on the same day. SQ-BSE developed, in addition to spongiform changes and astrogliosis, a complex proteinopathy with severe accumulations of PrP^TSE^, hyperphosphorylated tau (p-tau), ubiquitin, and α-synuclein but without the beta-amyloid (Aβ) protein typical of Alzheimer’s disease.

## 2. Results

### 2.1. Seven Squirrel Monkeys Inoculated with Material Containing BSE Agent Developed a Similar Neurological Disease (SQ-BSE) after Significantly Different Incubation Times Compared to Four Uninfected Squirrel Monkeys (SQ-Uninfected)

We previously showed that transgenic mice expressing bovine PrP (TgBo) inoculated with a bacteria-free filtrate of the same 1% C-BSE-infected brain suspension used to inoculate SQ developed neurological signs confirmed neuropathologically as TSE [11]. Those preliminary studies demonstrated that the original C-BSE brain extract used in the experiments described here contained infectivity (5 log_10_ IC ID_50_ per 0.03 mL). SQ were inoculated with serial dilutions of the same material used to inoculate TgBo mice. Ten years after inoculation the experiment was terminated.

Seven monkeys, designated SQ-BSE, developed neurologic signs typical of TSE, including loss of normal responsiveness (withdrawal), tremor, bradykinesia, jerky uncoordinated movements, and generalized weakness. No monkeys became noticeably irritable or aggressive and none lost weight. Three animals inoculated with 10^−1^ (10%) unfiltered low-speed clarified C-BSE agent were euthanized ~3.2 years (~38.4 months) after inoculation. Two animals inoculated with 10^−2^ unfiltered, low-speed clarified C-BSE suspension were euthanized 3.7 years (44.4 months) after inoculation. Two animals inoculated with the 0.45-µm-filtered bacteria-free 10^−2^ C-BSE-infected brain suspension also developed TSE. One of those animals (SQ-BSE 735) was euthanized with signs of TSE 3.3 years (39.6 months) after inoculation. SQ-BSE 735’s cage mate (736) had been inoculated by the same investigator with the same dilution and volume of C-BSE inoculum, on the same day and in the same facility used to inoculate all other animals described here; SQ-BSE 736 developed similar signs of neurological illness including weakness and ataxia, but almost five years (60 months) later (a total of 7.9 years [94.8 months] after inoculation). SQ-BSE 736’s neurological disease progressed over a period of 50 days until the animal was euthanized. Both SQ-BSE 735 and SQ-BSE 736 showed similar neurological signs and durations of overt illness (Table 1). All other animals inoculated with dilutions of BSE brain extract diluted > 10^−2^ remained asymptomatic until the experiment was terminated.

The unexpectedly large difference in incubation times between SQ-BSE 735 and SQ-BSE 736 prompted us to analyze the open reading frame (ORF) of the *PRNP* genes in SQs under investigation looking for genetic variants [12]. Direct sequence analysis showed that SQ-659, 707 and 718, having no TSE, as well as SQ-BSE 721, 722, 735, 737, 738, and 739, all having TSE with similar incubation times after inoculation (mean 38.5 months, standard deviation 5.2 months), and SQ-BSE 736, developing TSE after more than twice the incubation time (94.8 months) of its cage mate, all had identical *PRNP* nucleotide sequences. Therefore, differences in the open reading frame of the *PRNP* gene did not cause the longer incubation time of SQ-BSE 736.

### 2.2. PrP^TSE^ in SQ-BSE Brains

**Figure 1:**
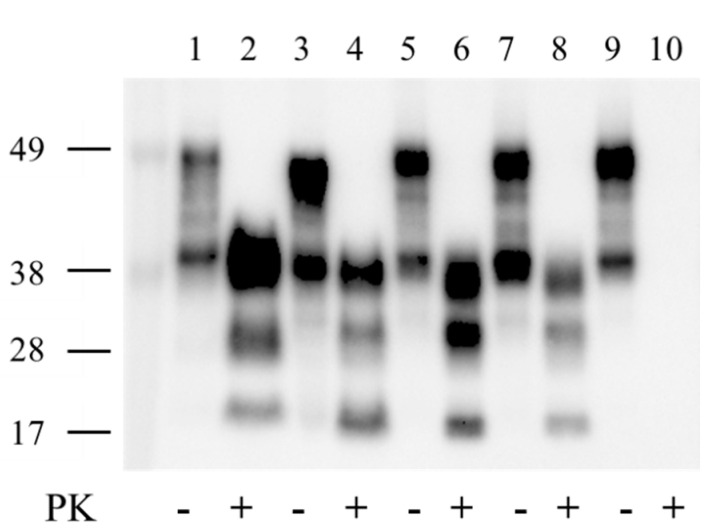
Western blot of brain extracts from bovine BSE (lanes 1–2), human vCJD (lanes 3–4), SQ-BSE 735 (lanes 5–6), SQ-BSE 736 (lanes 7–8) and from SQ 659 uninfected control (lanes 9–10). Total PrP (brain extracts with no proteinase K [PK] digestion) are shown in lanes 1, 3, 5, 7 and 9; brain extracts treated with PK are shown in lanes 2, 4, 6, 8 and 10. Western blots were probed with PrP monoclonal antibody 6D11.

Frozen brain tissue from SQ with neurological signs and SQ with no disease were tested by enzyme immunoassay (EIA) and western immunoblot. Brain extracts of ill animals with overt TSE, including SQ-BSE 735 and SQ-BSE 736, contained PrP^TSE^ detected by EIA. A western immunoblot of samples resolved in 12% SDS-PAGE was probed with anti-PrP Mab 6D11; samples from SQ-BSE 735 and SQ-BSE 736 showed identical PrP^TSE^ isoforms with prominent diglycosylated and monoglycosylated bands resistant to digestion with proteinase-K (Figure 1). In contrast, the PrP^TSE^ in the bovine C-BSE inoculum and in vCJD-infected human brain suspensions (World Health Organization Candidate Biologic Reference CJD Materials prepared in 0.3 mmol/L sucrose [13]) showed nonglycosylated PrP^TSE^ bands of 18 kDa for C-BSE and 17 kDa for vCJD compared to 16.5 kDa for SQ-BSE 735 and for SQ-BSE 736.

The electrophoretic mobilities of the nonglycosylated PrP^TSE^ isoforms in the inoculum (C-BSE) and human vCJD, different from those in all SQ-BSE, indicate that PrP^TSE^ isoforms with different major PK-cleavage sites accumulated in bovine C-BSE, human vCJD, and SQ-BSE.

Frozen brain tissue from all SQ without neurological signs were used for EIA analysis and confirmed negative for the presence of PrP^TSE^. Brains of four monkeys (SQ 659, 707, 718 and 723) with no signs of TSE were also confirmed to be negative by histopathologic, immunohistochemical and Western blot analyses. Those brains served as negative controls. Paraffin sections of spleen and liver from those negative controls and seven monkeys with clinical signs of TSE (SQ-BSE 721, 722, 735, 736, 737, 738 and 739) were probed with PrP monoclonal antibodies 6H4 and 3F4; brain from SQ 659 served as negative control and brain from SQ-BSE 739 as positive control. No PrP^TSE^ was observed in peripheral organs of any monkey tested. EIA with spleen was not successful.

### 2.3. Neuropathology of SQ-BSE

Monkeys inoculated with serially diluted C-BSE bovine brain extract and showing neurological disease all developed widespread spongiform degeneration (SD) of similar pattern and distribution: mild to moderate in the upper neuronal layers and severe in the deeper cell layers of the frontal and occipital cortices. SD was minimal or absent in the temporal cortex but moderate to severe in the basal ganglia, particularly in the thalamus and hypothalamus. SD was moderate in the CA1 region, while other areas of the hippocampus showed no obvious pathology. SD was also observed in brainstem and cerebellum, the latter also showing mild to moderate loss of granule cells.

Immunohistochemical analyses of SQ-BSE brains used both antibodies 3F4 and 6H4. Better results were obtained with 6H4 used for all subsequent experiments. PrP^TSE^ accumulated as moderate to severe fine-punctate, pericellular, linear, and coarse deposits, mostly adjacent to areas with SD. We observed a correlation between severity of SD and intensity of PrP^TSE^ deposition. This association between SD and PrP^TSE^ deposition was confirmed in the temporal cortices of all SQ-BSE, showing little or no vacuolation and minimal or absent PrP^TSE^. No plaque-like protein aggregates were seen in brain of any animal. Astrogliosis was most severe in the same areas showing SD and PrP^TSE^. Similar neuropathologic changes were seen in brains of all affected animals. However, the brain of SQ-BSE 736 showed more severe SD, PrP deposition, and gliosis than did brains of animals with shorter incubation times.

**Figure 2:**
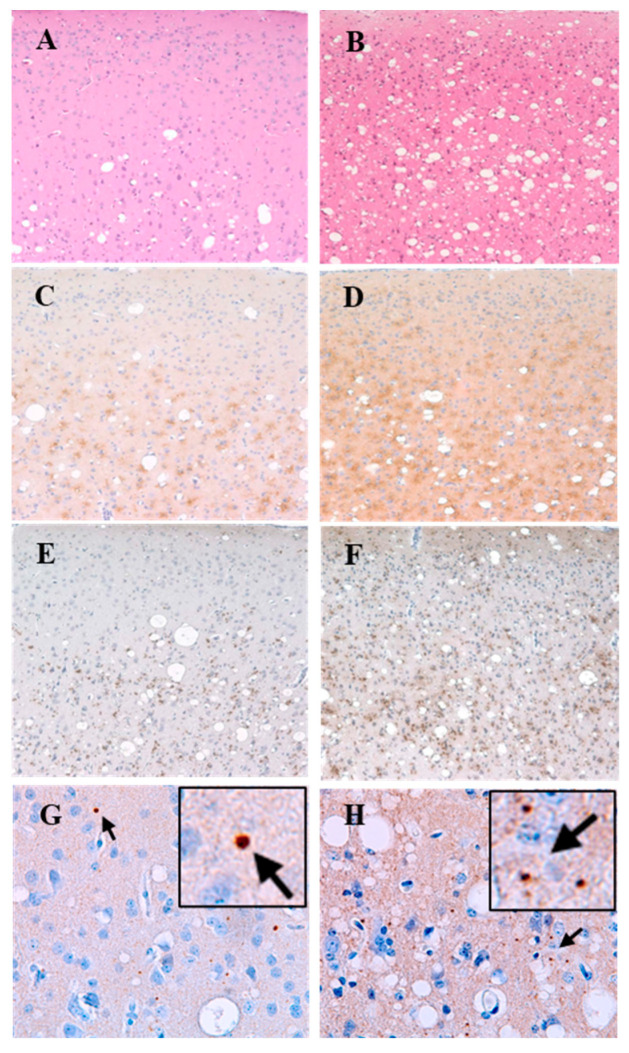
Comparative neuropathology of two SQ-BSE with extremely different incubation times. Squirrel monkeys inoculated with classical BSE (SQ-BSE) developed TSE and a complex proteinopathy (**A**–**H**). SQ-BSE 735, incubation period 3.3 years (39.6 months) (**A**,**C**,**E**,**G**); SQ-BSE 736 incubation period 8.1 years (94.8 months) (**B**,**D**,**F**,**H**). Moderate (**A**) or severe (**B**) spongiform degeneration in frontal cortex. Sections stained with hematoxylin-eosin. Moderate (**C**) or severe (**D**) PrP^TSE^ in frontal cortex. Moderate (**E**) or severe (**F**) p-tau immunopositivity in frontal cortex. (**A**,**B**) sections stained with hematoxylin-eosin; (**C**,**D**) sections immunostained with anti-PrP antibody 6H4; (**E**,**F**) sections immunostained with anti-tau antibody AT8. Panels A–F, 20× magnification. (**G**,**H**) sections of frontal cortex immunostained with 4D6 antibody against α-synuclein showing small granular accumulations (arrows), 40× magnifications and further enlarged in squared areas.

Brains of all SQ-BSE showed widespread accumulations of p-tau, forming dots, rods, and, in some brains, small circular deposits with unstained cores. Prominent p-tau deposits were seen in cerebral cortex, basal ganglia, cerebellum (molecular, Purkinje and granule cell layers), and in brain stem. The patterns and distribution of p-tau were similar in all animals with TSE. We also observed fine punctate deposits of α-synuclein in brains of all SQ-BSE, most severe in SQ-BSE 736 with similar patterns and distribution, most obvious in the same areas with SD, astrogliosis, and deposits of PrP^TSE^ and p-tau (Figure 2). Those proteins were not detected by immunohistochemistry in brain sections of uninoculated roughly age-matched SQ or in brain sections of SQ-BSE incubated with secondary antibody alone without exposure to primary antibodies. Therefore, we conclude that deposits of p-tau and α-synuclein accompanied those of PrP^TSE^ were constant and prominent neuropathologic features of SQ-BSE. The presynaptic marker synaptophysin was preserved in spared brain regions (data not shown), suggesting that neurodegeneration was selective.

## 3. Discussion

Although squirrel monkeys have long been recognized as susceptible to infection with several TSE agents [2,14] transmission of BSE into this species has not been reported by other research groups [15]. The results presented here, after a ten-year observation period, show that intracerebral and peripheral inoculation of C-BSE agent into SQ transmitted a neurological disease with progressive behavioral, motor and cerebellar signs typical of TSEs. Neuropathologic examinations of brains from all seven animals with experimental BSE showed similar severe spongiform degeneration in the cerebrum, cerebellum and brainstem. Abundant PrP^TSE^ deposits were present in most brain areas analyzed, closely correlated with severity of spongiform degeneration and astrogliosis. However, no obvious spongiform degeneration or PrP accumulation occurred in the temporal cortex; the hippocampus showed only minimal PrP^TSE^ accumulation in the CA1 area.

Brains of all animals with BSE showed severe tauopathy (in cerebral cortex, basal ganglia, cerebellum and brainstem) in the same areas with spongiform degeneration, PrP^TSE^, and astrogliosis, suggesting a correlation between neurodegeneration and complex protein accumulation in SQ-BSE. The morphology and distribution of p-tau deposits differ from those seen in humans with Alzheimer’s disease and with primary tauopathies [16]. However, p-tau rods similar to those described here have been described in other experimental TSEs, in humans with vCJD [16,17,18,19] and in a non-transmitted encephalopathy of UK cattle [20]. Our findings suggest that p-tau probably accumulated as a secondary event following appearance of spongiform encephalopathy and accumulation of PrP^TSE^.

While it would have been interesting to study early development of the several histopathologic changes and accumulation of proteins during the silent incubation period of experimental BSE, especially the temporal relationship in appearance and accumulation of PrP^TSE^ and p-tau, the study was not designed for that purpose and it was not feasible. Although p-tau is considered a neuropathologic hallmark in several degenerative diseases of the central nervous system, tau protein displays other post-translational modifications (e.g., glycosylation, acetylation), raising a possibility that other tau species might also participate in the disease process [21]. No obvious differences in distribution of PrP^TSE^ and p-tau deposits or in astrogliosis were observed in SQ-BSE after incubation periods ranging from 29 to 98.6 months or in those with different durations of clinical signs ranging from two to five months. We did find larger amounts of PrP^TSE^, astrogliosis, p-tau, and α-synuclein in the brain of SQ-BSE 736 compared with SQ-BSE 735, suggesting that more post-translationally modified proteins accumulated during the longer asymptomatic phase of BSE. The extended incubation period in SQ-BSE 736 underscores one limitation of rodent models expressing human PrP to assess pathogenesis of human later-onset TSEs—that most mice rarely live much longer than two years.

The severe pathology seen in the brainstem and the cerebellum of SQ-BSE is of special interest. Most patients become demented in the terminal stages of many neurodegenerative diseases; consequently, postmortem studies of their brains have usually directed special attention to lesions in the telencephalon (supratentorial) while rather neglecting degenerative changes in brainstem and cerebellum. Further studies should explore whether loss of cortical neurons in TSEs causes secondary infratentorial pathology or if infratentorial regions are also a primary target of disease. Additional studies might also determine to what extent the nuclei of the brainstem (essential to maintain vital neurological functions) affected by neurodegenerative processes in TSEs contribute to the rapid clinical decline typical of TSEs. Recent studies of early AD and PD also found considerable unexpected involvement of brainstem nuclei, findings that might profoundly change present concepts on origin, anatomical spread, and early clinical diagnosis of these diseases [22].

Previous studies suggest that a disturbed insulin signaling cascade may be implicated in the pathways through which soluble Aβ protein induces tau protein to phosphorylate [23]. Vasconcelos concluded that Aβ induced heterotypic seeding of tau filaments by spread of abnormal tau isoforms [24], possibly because hyperphosphorylation of tau, leads to self-assembly [25]. Here, we report that both p-tau and α-synuclein accumulated in all the same brain areas with neurodegeneration, but without forming detectable Aβ-amyloid plaques. Those findings suggest that subfibrillary PrP^TSE^ aggregates might have stimulated a complex proteinopathy involving both tau and α-synuclein proteins that actively contributed to pathogenesis, perhaps resulting from loss of some function of PrP^C^.

An incidental finding of significance in the study is that differences in incubation times of BSE in SQ cannot be attributed to variation in the open reading frames of *PRNP* genes because those sequences were identical in all monkeys tested; any genetic differences influencing incubation times must reside elsewhere in the genome. Furthermore, these results suggest caution before relying on similarities or differences in PrP^TSE^ glycotype to infer that a TSE infection in one species was acquired from a TSE agent originating in the same or another species, because PrP of three species infected with the classical C-BSE agent in our study displayed different glycotypes. In any case, results with the SQ-BSE model presented here affirm that primate models can improve our understanding of the pathogenesis of human neurodegenerative diseases.

## 4. Materials and Methods

### 4.1. BSE Agent and Bioassay

All studies involving samples and suspensions of tissue containing or potentially containing the BSE agent and animals inoculated with those materials were handled under biosafety-level-3 (BSL-3) and animal-BSL-3 (ABSL-3) procedures as described in the 5th and 6th editions of *Biosafety in Microbiological and Biomedical Laboratories* (https://www.cdc.gov/labs/pdf/CDC-BiosafetyMicrobiologicalBiomedicalLaboratories-2020-P.pdf (accessed on 12 May 2022)) in facilities with containment equipment inspected and approved by the United States Department of Agriculture (USDA). The BSE-infected bovine brain sample studied was a 10% suspension in 0.25-M sucrose (kindly provided under USDA permit by Dr. Torsten Seuberlich, NeuroCentre, National and OIE Reference Laboratories for BSE and Scrapie, Vetsuisse Faculty, University of Bern, Switzerland) was of the “classical” BSE type (C-BSE) originally described. Infectivity titer was determined in SQ purchased from Osage Research Primates (Kaiser, MO, USA) and housed in a USDA-approved BSL-3/ABSL-3 animal facility. All animal experiments were reviewed and approved by the US Food and Drug Administration Institutional Animal Care and Use Committee. Ten-fold serial dilutions of the BSE-infected brain were prepared (10^−1^ through 10^−9^) and 150-µL aliquots were injected through a burr hole into the left frontal region of the SQ brain; 150-µL aliquots were also inoculated intraperitoneally into the right lower quadrant of the abdomen. Three animals in each group were inoculated with BSE inocula diluted 10^−1^ through 10^−6^; two animals per group were inoculated with the same material diluted 10^−7^ through 10^−9^. In addition, a 10^−2^ (1%) dilution of the same BSE-infected brain suspension in saline was filtered through a pre-moistened 0.45-µm Millipore membrane to obtain a bacteria-free extract to inoculate cell cultures as previously described [11]; the 10^−2^ filtered brain suspension was bioassayed in two monkeys. Animals were inspected daily for signs of illness and euthanized promptly after neurological signs or serious intercurrent illness developed. Animals surviving for ten years or more without clinical disease were euthanized. After euthanasia, brains were removed and divided in half. The left cerebral hemisphere, cerebellum, and brainstem were fixed in formalin for histologic and immunohistochemical studies; the right sides were stored frozen for biochemical analyses. Animals, inocula, and related information are summarized in Table 1. 

### 4.2. Histopathological and Immunohistochemical Analyses and Enzyme Immunoassays

Frozen brain tissue was tested by Western blotting and enzyme immunoassays (EIA, HerdCheck BSE-Scrapie Ag Test [“IDEXX” Test], IDEXX Laboratories, Inc., Westbrook, ME, USA) to detect PrP^TSE^ using published protocols previously described [26]; proteins were separated in 12% SDS-PAGE, transferred to polyvinylidene fluoride (PVDF) membranes, and then probed with monoclonal antibody (Mab) 6D11 directed against PrP amino acids 93–109 (Santa Cruz Biotechnology, Santa Cruz, CA, USA). Histopathological and immunohistochemical studies used formalin-fixed, paraffin-embedded tissue sections and published protocols [10,27]. For immunohistochemical studies, sections were probed with anti-PrP Mab 3F4 (BioLegend, formerly Covance, San Diego, CA, USA) directed against human PrP amino acids 109–112, and Mab 6H4 (Prionics, Zurich, Switzerland) directed against human PrP amino acids 144–152. Astrogliosis was detected using a polyclonal antibody to bovine glial fibrillary acidic protein (GFAP, Dako North America, Carpinteria, CA, USA). Brain sections were also probed with Mab 4G8 directed against amino acids 17-24 of Aβ (BioLegend, formerly Covance, San Diego CA), Mab AT8 directed against doubly phosphorylated tau at amino acids Ser202 and Thr205 (Thermo Fisher Scientific, Rockford, IL, USA), and 4D6 antibody raised against recombinant human α-synuclein (Abcam, Waltham, MA, USA) and synaptophysin 1 (Synaptic Systems, Goettingen, Germany). Amounts of spongiform degeneration and PrP^TSE^ accumulations were scored semi-quantitatively by a commonly used four-point scale modified from Fraser and Dickinson [28] (0: none; 1: few/occasional, mild; 2: moderate; 3: severe) in the cortex, basal ganglia, cerebellum and brain stem of all animals. Formalin-fixed tissues from spleen and liver of selected animals were paraffin-embedded and sections used for immunohistochemical studies with anti-PrP antibodies 3F4 and 6H4. Deposits of α-synuclein were too small and sparse to score semi-quantitatively.

### 4.3. Prion Protein Gene (PRNP) Analysis

We sequenced the open reading frames of the *PRNP* gene in 12 SQ to identify any point mutations or polymorphisms using published protocols [29]. Total RNA was extracted from frozen brain samples of SQ with and without TSE (confirmed neuropathologically) to generate cDNA for sequencing (TRIzol^®^ Plus RNA Purification Kit, Thermo Fisher Scientific, Invitrogen, Leicestershire, UK). SQ brain samples were from three groups: (A) SQ 14 and 373, healthy control animals housed in a BSL-2 TSE-free facility; (B) SQ 659, 707, and 718, inoculated with cells exposed to BSE agent [11] and later euthanized due to intercurrent illness (or at the end of the study) having no neurological signs or histopathological changes of TSE and (C) SQ 721, 722, 735, 736, 737, 738, and 739, inoculated with C-BSE agent and confirmed by histopathology to have TSE. *PRNP* analyses were performed using mRNA purified on dT25 DynaBeads (Dynabeads^®^ mRNA DirectTM Purification Kit, Thermo Fisher Scientific) according to manufacturer’s instructions. cDNA was synthesized using the SuperScript IV First-Strand Synthesis System (Thermo Fisher Scientific). Two pairs of specific primers were used to amplify SQ cDNA (pairs A and B) that was then sequenced with primers SQMF2 and SQMR3:

**Pair A**

SQ/NN: 5′-TACTGAGAATTCATGGCGAACCTTGGCTACTGG-3′

SQ/SS: 5′-TACTGAGTCGACCCTTCCTCATCCCACTATCAGG-3′

**Pair B**

SQ52/74: 5′-TACTGGAATGCTGGTTCTCTTTG-3′

SQ730-752: 5′-TGCTCGATCCTCTCTGGTAATA-3′

**Sequencing primers**

SQMF2-TCATCATGGCGAACCTTGG

SQMR3-AGATGGTGGAAACAGGAAGAC

## Figures and Tables

**Table 1:** Clinical and pathological characterization of SQ-BSE.

	*SQ-Uninfected*				*SQ-BSE*						
*SQ-BSE Identification*	*659*	*707*	*718*	*723*	*721*	*722*	735 **	736 *	*737*	*738*	*739*
IP/Y(M)	n/a	n/a	n/a	n/a	3.5 (42)	3.8 (45.6)	3.3 (39.6)	8.1 (94.8)	3.1 (37.2)	3.1 (37.2)	2.4 (28.8)
CD/M	n/a	n/a	n/a	n/a	4	3	2	2	5	2	5
C-BSE 1	n/a	n/a	n/a	n/a	n/a	n/a	10^−1^	10^−1^	10^−1^	10^−1^	10^−1^
C-BSE 2	n/a	n/a	n/a	n/a	10^−2^	10^−2^	n/a	n/a	n/a	n/a	n/a
Vero	9 × 10^8^	n/a	n/a	n/a	n/a	n/a	n/a	n/a	n/a	n/a	n/a
R9ab	n/a	2 × 10^8^	n/a	n/a	n/a	n/a	n/a	n/a	n/a	n/a	n/a
MDCK	n/a	n/a	1.3 × 10^9^	n/a	n/a	n/a	n/a	n/a	n/a	n/a	n/a
HEK293	n/a	n/a	n/a	2 × 10^9^	n/a	n/a	n/a	n/a	n/a	n/a	n/a
ataxia	−	−	−	−	+	+	+	+	+	+	+
tremor	−	−	−	−	−	+	+	−	+	+	+
weakness	−	−	−	−	+	+	+	+	−	−	−
bradykinesia	−	−	−	−	+	−	−	+	+	+	+
myoclonus	−	−	−	−	+	−	+	−	+	+	−
SD	0	0	0	0	2–3	2–3	2–3	3	2–3	2–3	2–3
astrogliosis	0	0	0	0	2–3	2–3	2–3	3	2–3	2–3	2–3
PrP^TSE^	0	0	0	0	2–3	2–3	2–3	3	2–3	2–3	2–3
p-tau	0	0	0	0	2–3	2–3	2–3	3	2–3	2–3	2–3
α-syn	n/a	n/a	n/a	n/a	n/a	n/a	n/a	n/a	n/a	n/a	n/a

IP/Y(M), incubation period in years and in months in parenthesis. CD/M, duration of clinical disease in months. C-BSE 1, unfiltered low-speed clarified, 10^−1^ (10%) C-BSE infected bovine brain suspension. C-BSE 2, 0.45-µm-filtered bacteria-free, 10^−2^ (1%) C-BSE-infected bovine brain suspension. Vero, R9ab, MDCK, HEK293: cell lines exposed to C-BSE agent that did not become infected [11]. Numbers are estimated total numbers of cells injected into each monkey. PrP^TSE^, abnormal prion protein. p-tau, hyperphosphorylated tau protein. α-syn, α-synuclein detected with 4D6 antibody. The tiny sizes and sparse distribution of α-syn deposits were not amenable to semi-quantitative scoring. *, SQ-BSE 736 (long IP). **, SQ-BSE 735 (short IP). +, sign of illness noted. −, sign of illness not noted. SD, spongiform degeneration. n/a, not applicable. Astrogliosis, PrP^TSE^, and p-tau: aggregate semi-quantitative scores.

## Data Availability

The data produced in these experiments will be provided by the corresponding author on request.
